# Assessing Children’s Dietary Pesticide Exposure: Direct Measurement of Pesticide Residues in 24-Hr Duplicate Food Samples

**DOI:** 10.1289/ehp.1002044

**Published:** 2010-07-16

**Authors:** Chensheng Lu, Frank J. Schenck, Melanie A. Pearson, Jon W. Wong

**Affiliations:** 1 Exposure, Epidemiology, and Risk Program, Department of Environmental Health, Harvard School of Public Health, Boston, Massachusetts, USA; 2 U.S. Food and Drug Administration, Southeast Regional Laboratory, Atlanta, Georgia, USA (retired); 3 Department of Environmental and Occupational Health, Rollins School of Public Health, Emory University, Atlanta, Georgia, USA; 4 U.S. Food and Drug Administration, Center for Food Safety and Applied Nutrition, College Park, Maryland, USA

**Keywords:** children’s diets, dietary pesticide exposure, organophosphate pesticides, pesticide residue, pesticide risk assessment, pyrethroid insecticides

## Abstract

**Background:**

The data presented here are a response to calls for more direct measurements of pesticide residues in foods consumed by children and provide an opportunity to compare direct measures of pesticide residues in foods representing actual consumption with those reported by the U.S. Department of Agriculture Pesticide Data Program.

**Objective:**

We measured pesticide residues in 24-hr duplicate food samples collected from a group of 46 young children participating in the Children’s Pesticide Exposure Study (CPES).

**Methods:**

Parents were instructed to collect 24-hr duplicate food samples of all conventional fruits, vegetables, and fruit juices equal to the quantity consumed by their children, similarly prewashed/prepared, and from the same source or batch. Individual or composite food items were analyzed for organophosphate (OP) and pyrethroid insecticide residues.

**Results:**

We collected a total of 239 24-hr duplicate food samples collected from the 46 CPES children. We found 14% or 5% of those food samples contained at least one OP or pyrethroid insecticide, respectively. We measured a total of 11 OP insecticides, at levels ranging from 1 to 387 ng/g, and three pyrethroid insecticides, at levels ranging from 2 to 1,133 ng/g, in children’s food samples. We found that many of the food items consumed by the CPES children were also on the list of the most contaminated food commodities reported by the Environmental Working Group.

**Conclusions:**

The frequent consumption of food commodities with episodic presence of pesticide residues that are suspected to cause developmental and neurological effects in young children supports the need for further mitigation.

In the 1990s, a book published by the National Research Council (NRC) titled *Pesticides in the Diets of Infants and Children* (NRC 1993) and the passage of the Food Quality Protection Act of 1996 (FQPA 1996) were hailed as two major milestones in the United States in recognizing the need for systematic efforts in reducing pesticide exposure in vulnerable populations, especially infants and children. An important summary statement in the NRC report declared that “dietary intake represents the major source of pesticide exposure for infants and children, and the dietary exposure may account for the increased pesticide-related health risks in children compared with adults.” After the NRC report, the newly enacted FQPA aimed to fundamentally change U.S. policies in governing the acceptability of pesticide residues in foods and required the U.S. Environmental Protection Agency (EPA) to assure the public that there is a “reasonable certainty of no harm” from pesticide exposures for pregnant women, infants, children, and other vulnerable groups. Despite many years of effort in implementing the FQPA, monitoring and managing dietary pesticide exposures and risks are still an ongoing challenge. A recent report published by the U.S. Office of Inspector General (2006) has stressed the need for data-driven indicators of pesticide dietary exposures/risks.

The data presented here are in response to these calls for more direct measurements of pesticide residues in foods consumed by children and provide an initial assessment of the effects of the U.S. EPA’s actions on assessing dietary pesticide risks (U.S. Office of the Inspector General 2006). In this study, we measured pesticide residues in 24-hr duplicate foods from a group of elementary school–age children participating in the Children’s Pesticide Exposure Study (CPES). In addition, the CPES data provided an opportunity to compare direct measures of pesticide residues in foods representing actual consumption with measured values reported by the Pesticide Data Program [PDP; U.S. Department of Agriculture (USDA) 2009], a national database of pesticide residues found on foods that are ready for distribution in the United States. This comparison allows a unique examination of “real-world” applicability of the national data set.

## Materials and Methods

### Children’s Pesticide Exposure Study

CPES was initially conducted in the suburban Seattle, Washington, area (CPES-WA) from summer 2003 to winter 2004 and was later repeated in Atlanta, Georgia (CPES-GA), in 2006/2007 with the same study protocol. Twenty-three children 3–11 years of age were recruited for each CPES study from local public elementary schools and Montessori preschools. (One of the children who participated in CPES-GA study was 12 years old at the beginning of study.) Schools did not provide any assistance in recruiting subjects except for granting permission to send home with the children a letter and a fact sheet describing the study. Families that were interested in participating in this study then contacted the research group directly. A screening questionnaire was conducted over the phone to confirm eligibility, which included children exclusively consuming conventional (nonorganic) diets and spending most of their time in one residence, and the willingness of parents or caregivers to provide assistance in collecting specimen samples. Once a participant was enrolled, an in-house appointment was made to go over the study protocol and train the parents/caregivers on how to collect specimens and 24-hr duplicate food samples. All parents provided written informed consent, and all children provided written informed consent (if ≥ 10 years of age) or oral assent (< 10 years of age) before data collection began. A questionnaire was also administered by the research staff during this appointment that asked about household pesticide use to account for nondietary sources of pesticide exposure.

Each CPES child agreed to participate in multiple consecutive days of sampling in four seasons over a 12-month period (four seasons). They also participated in a 5-day organic diet intervention in summer and fall seasons for CPES-WA children and summer and winter seasons for CPES-GA children in which organic groceries were provided to replace foods (mostly fresh fruits, vegetables, grains, and fruit juices) that their child usually ate (Lu et al. 2006a, 2006b). In additional to the 24-hr duplicate food samples (described below), two spot urine samples and dietary consumption information were collected daily from all CPES children. The University of Washington Human Subject Division (no. 03-5899) and Emory University Internal Review Board (no. 084-2005) approved the use of human subjects in this study and continued data analysis.

### Food sample collection and process

In both CPES studies, 1 day’s worth of 24-hr duplicate food samples were collected by parents and children on a study day selected at their convenience on which the child-participant consumed nonorganic foods in the summer and fall seasons for CPES-WA children, and summer and winter for CPES-GA children. Families were instructed to provide nonorganic samples of all fruits, vegetables, and fruit juices that were equal to the quantity consumed by the child, similarly prewashed/prepared, and obtained from the same source or batch (i.e., if the child ate half an apple, the parents submitted the other half of the apple, or if the child ate the whole apple, the parent submitted a second apple from the same batch/bag of apples). Individual food items were collected using zip-lock bags, labeled, and stored in families’ refrigerators until picked up by the research staff the next day. Upon returning to the lab, individual food samples were weighed, homogenized, transferred to a condiment cup, and then frozen at −20°C until analysis at the Food and Drug Administration’s (FDA) laboratories. To ensure sufficient quantity for analytical analysis, food samples had to weigh at least 50 g. Individual food samples that weighed < 50 g were combined and processed as composite samples with other food items from the same meal. If a composite sample from a single meal weighed < 50 g, it was combined with food items from one or more additional meals to achieve a combined weight ≥ 50 g. We collected and analyzed 3 days’ worth of 24-hr duplicate food samples from CPES-WA children (two from summer and one from fall) and 2 days’ worth of 24-hr duplicate food samples from CPES-GA children (one each from summer and winter).

We also conducted a market basket analysis (MBA) to quantify organophosphate (OP) and pyrethroid insecticide residues in food items that CPES-WA children commonly consumed (based on daily dietary consumption information). We purchased 40 fresh fruit and vegetable items from one of the two neighborhood supermarkets where CPES-WA was taking place. The MBA food samples were processed identically and analyzed in the same FDA labs as the 24-hr duplicate samples. We did not conduct an MBA for CPES-GA children because of a lack of research resources.

### Chemical analysis

Homogenized food samples were extracted using a modified QuEChERS (quick, easy, cheap, effective, rugged, and safe) method (Schenck et al. 2009) originally developed for rapid multiresidue determination of OP insecticides in fresh fruits and vegetables at the 1-ng/g level (Anastassiades et al. 2003; Podhorniak et al. 2001; Wong et al. 2007). Briefly, this method entails salt-out acetonitrile extraction of food samples with magnesium sulfate and sodium chloride, followed by a solid-phase dispersive cleanup using graphitized carbon black and primary secondary amine solid-phase extraction sorbents and the addition of toluene. Extracts were evaporated almost to dryness under a nitrogen stream at 50°C and reconstituted in toluene for determination of OP and pyrethroid pesticide residues. The OP residues were determined using a gas chromatographic method with pulsed flame photometric detection, flame photometric detection, and mass spectrometry with limits of detection (LODs) ranging from < 1.0 to 10 ng/mL. The pyrethroid insecticide residues were determined using a gas chromatography method with halogen-specific detectors and/or mass spectrometric detectors, with LODs ranging from 5.0 to 25 ng/mL (Wong et al. 2010).

Fortification (quality assurance/quality control studies were performed by adding 1-, 10-, and 100-ppb levels of pesticides to four different pesticide-free commodities (grape, orange, spinach, and tomato). Recoveries ranged from 63% to 125%, with > 80% being achieved for most of the pesticides tested in each commodity (Schenck et al. 2009). For the 24-hr duplicate food samples that are analyzed, we analyzed individual isomers of pyrethroid insecticides (*cis*- and *trans*-permethrin) and then added them and reported the total concentrations. We did so for permethrin, cypermethrin, and esfenvalerate, but not for bifenthrin, which does not consist of isomers. The 24-hr duplicate food samples containing banana were not analyzed in the lab because of a problem with emulsion.

## Results

We analyzed a total of 110 and 129 24-hr duplicate food samples collected from 23 CPES-WA and 23 CPES-GA children, respectively. Table 1 shows the food consumption, mostly fruits, vegetables, and fruit juices, during the 24-hr duplicate food sampling days (3 days for CPES-WA and 2 days for CPES-GA). Consumption of certain food items differed between the two study groups, possibly because of differences in seasonal availability and the geographical distribution of certain food items between the states of Washington and Georgia. Among the top 12 most frequently consumed fruits, vegetables, and juices, seven were common to both cohorts: apples, apple juice, bananas, carrots, orange juice, peaches, and watermelon.

We found that 23% and 15% of the 24-hr duplicate food samples collected from CPES-WA and CPES-GA, respectively, contained either OP or pyrethroid insecticide residues (Table 2). The overall frequency of detection of pesticide residues (19%) in the 24-hr duplicate food samples was less than the frequency of detection in the MBA, in which 28% of produce purchased from a local supermarket contained either OP or pyrethroid insecticide residues. Among the 239 24-hr duplicate food samples that we collected, we found that 14% contained at least one OP pesticide and 5% contained at least one pyrethroid insecticide. We detected OP residues more often in the CPES-WA samples than in the CPES-GA samples (21% vs. 9%), but pyrethroid insecticide residues more often in CPES-GA than in CPES-WA samples (8% vs. 3%). Approximately 8% and 5% of the 24-hr duplicate food samples collected from CPES-WA and CPES-GA participants, respectively, contained multiple OP or pyrethroid insecticide residues.

Tables 3 and 4 show the concentrations of OP and pyrethroid insecticide residues in the 24-hr duplicate food samples collected from CPES-WA and CPES-GA children, respectively. Notably, we detected 11 OP and 3 pyrethroid insecticides in children’s food samples; levels ranged from 1 to 387 ng/g for OP and 2 to 1,133 ng/g for pyrethroid insecticides.

In general, the pesticide residues that we measured in CPES were within the ranges reported by the national PDP in 2004 (for comparison with CPES-WA samples collected in 2004) and 2006 (for comparison with CPES-GA samples collected in 2006) (Table 5). However, there were some exceptions. Several food samples collected from CPES-WA children contained higher residue levels than reported by the PDP, including *a*) a composite sample of cherries, a nectarine, and an apple with 387 ng/g phosmet and 1 ng/g chlorpyrifos; *b*) a nectarine with 252 ng/g phosmet; *c*) a composite sample of raspberries, blueberries, blackberries, baby carrots, and a peach with 4, 7, and 45 ng/g malathion, phosmet, and azinphos-methyl, respectively; *d*) an apple with 111 ng/g azinphos-methyl; and *e*) a composite sample of lettuce, broccoli, and mushroom with 1,133 ng/g cypermethrin. In addition, two strawberry samples in the CPES-WA MBA (Table 6) contained 363 ng/g chlorpyrifos and 93 ng/g bifenthrin. Several food samples collected from CPES-GA children also contained higher residue levels than reported by the PDP (Table 5), including *a*) a composite sample of strawberries and spinach with 921 ng/g permethrin; *b*) a composite sample of potato, yellow bell pepper, broccoli, and ketchup with 10 ng/g chlorpyrifos, 9 ng/g methamidophos, 90 ng/g acephate; and 8 ng/g cypermethrin; and *c*) a celery sample with 78 ng/g methamidophos and 350 ng/g acephate. In addition, an apple sample provided by a CPES-WA child contained 111 ng/g azinphos-methyl, which exceeded the highest azinphos-methyl concentration reported by the PDP in 2004 (in pears, 42 ng/g). Similarly, a composite food sample with lettuce, broccoli, and mushroom contained cypermethrin levels that exceeded the highest levels reported by the PDP in 2004 (in lettuce and canned spinach, 90 ng/g). An MBA sample of strawberries contained 363 ng/g chlorpyrifos, which is close to the highest level that PDP reported in 2004 (in bell peppers, 470 ng/g). Commodities such as apples, bell peppers, celery, cherries, green beans, peaches, spinach, and strawberries that are frequently consumed by CPES children were often found by the PDP to have OP and pyrethroid pesticides in both 2004 and 2006 (Tables 1 and 5).

## Discussion

Characterizing dietary pesticide exposures, particularly for infants and children, has become an essential component of cumulative pesticide risk assessment, as mandated by the 1996 FQPA. Several reports (Lu et al. 2006a, 2006b, 2008, 2009; Schettgen et al. 2002) have clearly demonstrated the significant contribution of dietary intakes to the overall OP and pyrethroid pesticide exposure in children and highlighted the critical need to quantify the health risks associated with the low but chronic daily exposures to those pesticides. As the result of the 1993 NRC report, the U.S. Congress funded the PDP under the USDA to test pesticide residues annually in foods consumed most often by children and, to the extent possible, “as eaten.” Since its inception, PDP has tested > 200,000 food commodity samples for an extensive list of pesticides. When pesticide residues reported in the PDP database are combined with food consumption information, such as those surveyed in the Continuing Survey of Food Intakes by Individuals [now integrated in the National Health and Nutrition Examination Survey (NHANES) conducted by the Centers for Disease Control and Prevention] or the Total Diet Study conducted by the FDA (Egan et al. 2007), mathematical model simulations can be used to provide a basis for estimating pesticide dietary exposures and risks. In theory, those estimated pesticide intakes would reflect dietary exposures at the population level; however, it is often the consumption of so-called high-risk food commodities containing elevated pesticide residues that shapes the outcome of dietary risk assessment. Therefore, it is imperative to measure pesticide residues in foods actually consumed by children as part of their customary diets. Direct measures remove many of the unknowns and assumptions that must be made when calculating dietary risk assessments from separately collected consumption surveys and pesticide residue databases.

Before further interpreting the results obtained from the CPES, the limitations of this study should be acknowledged. First of all, many of the 24-hr duplicate food samples that we analyzed were composite food samples, so it is not possible to link pesticide residues found in the composite samples to individual food commodities. Second, the reported pesticide residues for those composite food samples may be underestimated because of the nature of compositing multiple food items into one sample. It is likely that we combined foods containing no pesticide residues with foods with pesticide residues, resulting in a diluted composite sample. Third, the 24-hr duplicate food samples collected from CPES-WA had been frozen at −20°C since 2004 before they were analyzed in 2007/2008. Although we anticipate some degradation of pesticides in certain food samples that undoubtedly would underestimate the true pesticide residues, it is difficult to quantify the magnitude. Fourth, the data reported here are limited by the small number of food samples that we collected from a total of 46 children on 5 different days across two seasons, and cannot be generalized to the overall population of children. Finally, although the collection of 24-hr duplicate food samples has been a common research method used to provide surrogate measurements of dietary pesticide exposure (Bradman et al. 2007; Fenske et al. 2002; MacIntosh et al. 2001; Melnyk et al. 1997), it is not known whether the 24-hr duplicate food samples provide valid estimates of the true levels of pesticide residues in foods actually consumed by children.

Overall, the levels of pesticide residues detected in the CPES food samples were similar to levels reported by the PDP; however, two CPES food samples and one MBA item exceeded reported PDP levels. These two CPES food samples probably represented a large single exposure for each child. The fact that two CPES children had a single exposure exceeding the PDP reported data highlights both the challenge in monitoring and managing dietary pesticide risks and the need to improve the PDP approach to more accurately capture real-world exposures. In fact, our comparison with the PDP data set was limited by the commodities that the PDP included in their reports. In both CPES study years, the PDP data analyzed only approximately one-third of the foods consumed by the CPES children. Although the PDP is the most comprehensive data set representing pesticide food residues in the United States, it does not test all foods, particularly foods that are commonly consumed by children, despite its increased focus on children’s foods in response to the FQPA. Furthermore, many commonly consumed foods are not tested every year by the PDP. These limitations threaten the success of conducting representative dietary pesticide exposure, risk assessments in infants and children (as mandated by FQPA), and trend analysis of dietary exposure risks.

The PDP database provides a basis for estimating pesticide dietary exposures and risks when combined with food consumption information and information on the toxicological potency of individual pesticides. This practice has been adapted by the Environmental Working Group (EWG) in their annual Shopper’s Guide to Pesticides reports (EWG 2009), in which commonly consumed fruits and vegetables, surveyed by the Total Diet Study, are ranked based on the pesticide residue data published by the PDP. As stated by EWG, the philosophy behind the Shopper’s Guide is to “give consumers the information they need to make choices in order to reduce pesticides in their diets.” Instead of conducting complex dietary pesticide risk assessments, the Shopper’s Guide provides a qualitative comparison on the overall load of pesticides found on commonly eaten fruits and vegetables. We found that 5 and 6 of the top 12 most consumed food commodities by CPES-WA (apple, peach, grape, carrots, and lettuce) and by CPES-GA children (strawberries, apple, pear, carrots, spinach, and peach), respectively, are among the top 12 most contaminated food items ranked by the Shopper’s Guide’s published in 2009. In fact, all of the top 12 most contaminated food items listed in the Shopper’s Guide’s were consumed at different frequencies by the CPES children. Because some of the high-pesticide-risk commodities, such as peaches, grapes, and pears, are usually not available to the consumers year round, seasonal variations in dietary pesticide exposures and risks may not be captured by risk assessors if seasonality is not taken into account when defining common dietary consumption patterns. We found support for this hypothesis in a recent study in which we found that CPES children consumed more seasonal food commodities, such as apples, peaches, nectarines, melon, grapes, pears, and strawberries, than did NHANES children in the same age range (Riederer et al. 2009). For food without seasonality, such as cow’s milk, apple juice, and orange juice, consumption differences were not apparent. Our comparisons illustrated how food consumption data collected in a cross-sectional manner, such as those of the NHANES, may not adequately capture seasonal variability in children’s dietary habits, and those measurement errors will no doubt be carried forward to the risk assessment steps.

Among the 44 duplicate food samples containing detectable pesticide residues, we found 11 OP and 3 pyrethroid insecticides, and 16 of these duplicate food samples (7%) contained more than one pesticide (OP or pyrethroids). Among the food samples with detectable pesticide residues, we found much higher levels of pyrethroid insecticides than OP pesticides. This finding is consistent with the tolerances for pyrethroid insecticides that are generally higher than for OP insecticides, and with the residues reported by PDP (Table 5). In addition, the higher pyrethroid insecticide residues may have resulted from excessive use of pyrethroid insecticides in the field to battle insect resistance problems. OP pesticide residues were detected more often in the CPES-WA food samples, whereas pyrethroid pesticides were detected more often in the CPES-GA food samples. The possible explanations for this finding may stem from contamination of foods resulting from residential use (Melnyk et al. 2009) of pyrethroid insecticides in the South/Southeast region, regional differences in agricultural practices, or changes in pesticide use over time. If this pattern holds true, it suggests that it is necessary to conduct dietary pesticide exposure and risk assessments based on geographic location. In addition to the seasonal nature of food consumption patterns among CPES children as reported in our recent study (Riederer et al. 2009), the findings of multiple pesticide residues in single food commodities and the geographical differences in the type of pesticides further demonstrate the complexity of conducting overall dietary pesticide exposure and risk assessments.

Although the pesticide residue levels measured in CPES were all below U.S. EPA tolerance levels (U.S. EPA 2010), it should be acknowledged that tolerances were established at on a per chemical per crop basis in order to ensure the best practice of pesticide uses in the field. Also, tolerance levels are intended for monitoring residues in raw produce at the farm gate, before washing, shipping, storage, marketing, and food preparation (Olden and Guthrie 2000). Furthermore, those tolerance levels do not consider exposure to multiple pesticides with common mechanisms of toxicity, such as the OP pesticides. Regardless, the findings from this study could be used for the basis of future regulatory decisions in terms of implementing a routine pesticide residue monitoring program within the PDP focusing on food items that are commonly consumed by children and on pesticides that are routinely detected in those foods. These data, along with seasonal consumption patterns, would be very useful in dietary pesticide risk assessment. In addition, the findings from this study, along with other reports (e.g., EWG’s Shopper’s Guide), could be used by parents and caregivers who want to keep nutritional foods in their children’s diets but avoid the intake of pesticide residues in the high-pesticide-risk items.

## Conclusions

The CPES is among the first to assess young urban/suburban children’s dietary pesticide exposures using the duplicate diet sampling methodology. We found that 44 of 239 (19%) 24-hr duplicate food samples contained at least one pesticide, and among the 44 food samples with detectable pesticide residue, 12 of them (27%) contained more than one pesticide. Although CPES pesticide residue levels were within the ranges of PDP data for corresponding years, there were a few exceptions in which the residue levels exceeded the highest values reported by PDP. We found that many of the food items consumed by the CPES children were also on the list of the most contaminated food commodities reported by the EWG. The observed seasonal and geographical differences in the type of pesticides and residues found in commonly consumed food items will affect dietary exposure and risk assessments and should be taken into account in risk assessments. Although none of the pesticide residues measured in the CPES exceeded the U.S. EPA’s tolerances, the frequent consumption of certain food commodities with episodic presence of pesticides that are known to cause developmental and neurological effects in young children underlies the need for further mitigation and should be monitored routinely by PDP. These much needed yearly pesticide residue data can be used not only to assess the impact of future regulatory policies on agricultural practices of pesticide uses but also to assess dietary exposures and the subsequent health risks resulting from dietary intakes.

## Figures and Tables

**Table 1:** The consumption of fresh produce and fruit juices by CPES-WA and CPES-GA children.

Food item	No. of samples^a^	Frequency of consumption (%)^b^
CPES-WA		
Orange juice	16	14.5
Blueberries	8	7.3
Watermelon	7	6.4
Apple^c^	7	6.4
Peach^c^	6	5.5
Lemonade	6	5.5
Grape^c^	6	5.5
Corn	6	5.5
Banana	6	5.5
Apple juice	6	5.5
Carrots^c^	5	4.5
Lettuce^c^	4	3.6
Broccoli	4	3.6
Apple sauce	4	3.6
Strawberries^c^	3	2.7
Raspberries	3	2.7
Peas	2	1.8
Potato	2	1.8
Pineapple	2	1.8
Nectarine^c^	2	1.8
Mushrooms	2	1.8
Honeydew	2	1.8
Grape juice	2	1.8
Cherries^c^	2	1.8
Spinach^c^	1	0.9
Onion	1	0.9
Mango	1	0.9
Green pepper^c^	1	0.9
Green beans	1	0.9
Grapefruit	1	0.9
Fruit punch	1	0.9
Celery^c^	1	0.9
Cauliflower	1	0.9
Cantaloupe	1	0.9
Asian pear	1	0.9
CPES-GA		
Banana	14	10.9
Strawberries^c^	13	10.1
Apple^c^	10	7.8
Orange juice	9	7.0
Peanut butter	7	5.4
Tomato	6	4.7
Watermelon	5	3.9
Pear^c^	5	3.9
Carrots^c^	5	3.9
Apple juice^c^	5	3.9
Spinach^c^	3	2.3
Peach^c^	3	2.3
Onion	3	2.3
Mushrooms	3	2.3
Lettuce^c^	3	2.3
Grape juice	3	2.3
Broccoli	3	2.3
Peas	2	1.6
Olives	2	1.6
Lemonade	2	1.6
Grape^c^	2	1.6
Fruit punch	2	1.6
Corn	2	1.6
Blueberries	2	1.6
Apple sauce	2	1.6
Potato	2	1.6
Raspberries	1	0.8
Plum	1	0.8
Mango	1	0.8
Green pepper^c^	1	0.8
Green beans	1	0.8
Celery^c^	1	0.8
Cantaloupe	1	0.8
Cabbage	1	0.8

a Consumption information was recorded from the days when 24-hr duplicate food samples were collected, and number of samples for each food item was summed among all children in each study.

b CPES-WA consumption frequency was calculated based on a total of 110 food samples (some of the samples were composited samples). CPES-GA consumption frequency was calculated based on a total of 129 food samples (some of the samples were composite samples).

c One of the 12 most pesticide-contaminated fruits and vegetables surveyed in 2008–2009 (EWG 2009).

**Table 2:** The frequency of pesticide residue detection in CPES 24-hr duplicate food samples.

Study	Total food samples collected	No. of samples with pesticide residue detected (%)	No. of food samples contaminated (%)			
			≥ 1 OP	≥ 1 pyrethroid	> 1 pesticide	> 2 pesticides
CPES-WA	110	25 (22.7)	23 (21)	3 (3)	9 (8)	3 (3)
CPES-GA	129	19 (14.7)	11 (9)	10 (8)	7 (5)	1 ( < 1)
MBA	40	11 (27.5)	9 (23)	2 (5)	1 (3)	0 (0)

**Table 3:** Twenty-four–hour duplicate fresh produce and fruit juice samples with detectable OP and pyrethroid insecticide residues collected from CPES-WA children.

Study season	Sample ID	Duplicate food item	Pesticide residue (ng/g)	
			OP	Pyrethroid
Summer 2003	1	Strawberries,^a^ green beans, dried raspberries, blueberries	Malathion (8)	Bifenthrin (149)
	2	Strawberries^a^	Malathion (4)	
	3	Cantaloupe	Malathion (11)	
	4	Apple sauce	Phosmet (23)	
	5	Apple sauce	Azinphos-methyl (13)	
	6	Peach,^a^ pineapple, apple sauce, sugar pea pod, corn	Omethoate (12) Dimethoate (10) Ethion (4)	
	7	Red grape,^a^ watermelon	Phosmet (18)	
	8	Cherries,^a^ nectarine,^a^ apple^a^	Phosmet (387) Chlorpyrifos (1)	
	9	Watermelon, apple^a^	Azinphos-methyl (14)	
	10	Nectarine^a^	Phosmet (252)	
	11	Raspberries, blueberries, blackberries, baby carrot,^a^ peach^a^	Malathion (4) Phosmet (7) Azinphos-methyl (45)	
	12	Cranberry juice	Acephate (2)	
	13	Cranberry juice	Acephate (1)	
Fall 2003	1	Apple^a^	Azinphos-methyl (111)	
	2	Lettuce,^a^ broccoli, mushrooms		Cypermethrin (1,133)
	3	Apple^a^	Phosmet (9) Azinphos-methyl (8)	
	4	Spinach, cauliflower		Permethrin (90)
	5	Asian pear	Phosmet (36)	
	6	Apple juice	Azinphos-methyl (10)	
	7	Orange juice	Chlorpyrifos (1)	
	8	Peas, corn	Dimethoate (6)	
	9	Juice box	Methamidophos (1)	
	10	Celery,^a^ onion, canned corn	Acephate (18)	
	11	Orange juice	Ethion (24)	
	12	Cranberry-apple juice	Acephate (3)	

Composite samples were analyzed if individual food items weighed < 50 g.

a One of the 12 most pesticide-contaminated food items surveyed in 2008–2009 (EWG 2009).

**Table 4:** Twenty-four–hour duplicate fresh produce and fruit juice samples with detectable OP and pyrethroid insecticide residues collected from CPES-GA children.

Study season	Sample ID	Duplicate food items	Pesticide residue (ng/g)	
			OP	Pyrethroid
Summer 2005	1	Strawberries,^a^ blueberries, grape juice		Bifenthrin (28)
	2	Potato	Phosalone (55)	
	3	Frozen berries (blue, black, raspberries), baby carrots,^a^ apple,^a^ banana	Phosmet (3) Chlorpyrifos (2)	
	4	Broccoli (cooked)		Permethrin (82)
	5	Peach^a^	Phosmet (86) Phosalone (29)	
	6	Green beans	Methamidophos (11) Acephate (39)	
	7	Watermelon, grape,^a^ cantaloupe	Phosalone (69)	
	8	Strawberries^a^		Bifenthrin (7)
	9	Strawberries^a^		Bifenthrin (2)
	10	Watermelon	Phosalone (83)	
	11	Carrots, baby green lettuce^a^	Phosalone (84)	Permethrin (58)
Winter 2006	1	Strawberries,^a^ spinach		Permethrin (921)
	2	Topping olives, mushroom, spinach		Permethrin (98)
	3	Orange	Phosmet (25) Chlorpyrifos (3)	
	4	Apple^a^		Permethrin (4)
	5	Apple,^a^ green salad	Phosmet (30)	
	6	Potato, bell pepper,^a^ broccoli, ketchup	Chlorpyrifos (10) Methamidophos (9) Acephate (90)	Cypermethrin (8)
	7	Celery^a^	Methamidophos (78) Acephate (350)	
	8	Spinach, tomato, spaghetti sauce		Cypermethrin (18)

Composite samples were analyzed if the single food items did not constitute > 50 g.

a One of the 12 most pesticide-contaminated food items surveyed in 2008–2009 (EWG 2009).

**Table 5:** Comparison of pesticides, residue levels, and commodities reported in CPES and PDP.

Pesticide^a^	Detected concentration range (ng/g) and sample containing the highest pesticide level			
	CPES-WA	PDP 2004	CPES-GA	PDP 2006
Acephate	Celery, onion, and corn (1–18)^b^	Green beans (2–2,000)	Celery (39–350)	Green beans (3–2,100)
Azinphos methyl	Apple (8–111)	Pear (13–42)	ND	Peach (2–620)
*Bifenthrin*	Strawberries, green beans, raspberries, and blueberries (93–149)^b^	Strawberries (5–300)	Strawberries, blueberries, and grape juice (2–28)^b^	Green beans (5–180)
Chlorpyrifos	Strawberries (1–363)^c^	Bell peppers (1–470)^c^	Potato, bell pepper, broccoli, and ketchup (2–10)^b^	Collard greens (2–6,300)^c^
*Cypermethrin*	Lettuce, broccoli, and mushrooms (1,133)^b^	Lettuce and canned spinach (25–90)	Spinach, tomato, spaghetti sauce (8–18)^b^	Collard greens (50–5,000)
Dimethoate	Peach, apple sauce, pineapple, pea pod, and corn (6–10)^b^	Bell pepper (3–35)	ND	Green beans (2–1,600)
*Esfenvalerate*	Cherries (71)^d^	ND	ND	Tomato (14–120)
Ethion	Orange juice (4–24)	ND	ND	ND
Malathion	Cantaloupe (4–11)	Lettuce (1–5,000)	ND	Celery (3–610)
Methamidophos	Juice box (1)^d^	Green beans (2–560)	Celery (9–78)	Green beans (2–500)
Omethoate	Peach, apple sauce, pineapple, pea pod, and corn (12)^b^,^d^	Lettuce and bell pepper (4–97)	ND	Kale greens (4–220)
*Permethrin*	Spinach and cauliflower (90)^b^,^d^	Canned spinach (48–11,000)	Spinach and strawberries (4–921)^b^	Kale greens (62–8,400)
Phosalone	ND	Apple (10–57)	Carrots and baby green lettuce (29–84)^b^	ND
Phosmet	Cherries, nectarine, and apple (9–387)^b^	Pear (3–1,600)	Peach (25–86)	Peach (5–2,200)

ND, not determined. CPES-WA data included samples from the MBA. PDP 2004 data are from USDA (2004). PDP 2006 data are from USDA (2006).

a Entries in italics are pyrethrins; all other entries are OP pesticide.

b Samples were composite food samples.

c Pesticide concentration exceeded the EPA tolerance level: 50 ppb chlorpyrifos (U.S. EPA 1996).

d Only one sample analyzed.

**Table 6:** MBA of OP and pyrethroid insecticide residues in fresh produce and fruit juice items commonly consumed by the CPES-WA children.

		Pesticide residue (ng/g)	
Study season	Food content	OP	Pyrethroid
Summer 2003	Cherries^a^		Esfenvalerate (71)
	Honeydew melon	Methamidophos (3)	
	Grape^a^	Methamidophos (1)	
	Nectarine^a^	Chlorpyrifos (1)	
		Methamidophos (2)	
	Strawberries^a^	Chlorpyrifos (363)	
	Lemonade	Methamidophos (trace)	
	Strawberries^a^		Bifenthrin (93)
Fall 2003	Orange	Ethion (1)	
	Strawberries^a^	Chlorpyrifos (3)	
	Peach^a^	Methamidophos (12)	
	Grape^a^	Chlorpyrifos (22)	

All the food samples were purchased in Washington State between 2003 and 2004.

a One of the 12 most pesticide-contaminated food items surveyed in 2008–2009 (EWG 2009).
